# Indication for Endoscopic Resection of Submucosal Colorectal Carcinoma: Special Reference to Lymph Node Metastasis

**DOI:** 10.1155/DTE.6.101

**Published:** 2000

**Authors:** Osamu Tsuruta, Yuichiroh Tsuji, Hiroshi Kawano, Shiroh Miyazaki, Masahide Watanabe, Keita Nakahara, Hideo Tateishi, Mitsutake Fujita, Shigeki Ban, Michio Sata, Atsushi Toyonaga, Minoru Morimatsu

**Affiliations:** 1 Department of Medicine II Kurume University School of Medicine 67 Asahi-machi Kurume City 830-0011 Japan; 2 Department of Pathology Kurume University School of Medicine 67 Asahi-machi Kurume City 830-0011 Japan

## Abstract

We investigated the relationship between histological factors and lymph node metastasis in 77 lesions with submucosally invasive colorectal carcinomas to establish useful criteria for lesions in which endoscopic treatment alone results in cure of malignancy. There were positive correlations between histological factors, including the level of invasion, the histologic grade, presence or absence of lymphatic invasion, presence or absence of budding, and lymph node metastasis (*p* < 0.05, *p* < 0.05, *p* < 0.005, *p* < 0.01). The presence or absence of venous invasion did not influence lymph node metastasis. Laparoscopic surgery involving lymph node dissection should be indicated for sm1 carcinoma lesions with unfavorable histological factors. In lesions diagnosed as sm2 or sm3 prior to resection, intestinal resection involving lymph node dissection by laparoscopic surgery should be directly performed without endoscopic resection.

In treating submucosally invasive colorectal carcinomas, the level of invasion can be clinically diagnosed, consequently endoscopic resection should be initially performed when lesions are evaluated as sm1 prior to resection. When histological investigation reveals sm1 carcinoma with histologic grade I (well-differentiated) or II (moderately-differentiated), and the absence of lymphatic invasion and budding, endoscopic treatment alone is sufficient.


*Diagnostic and Therapeutic Endoscopy*, Vol. 6, pp. 101–109
Reprints available directly from the publisher
Photocopying permitted by license only

© 2000 OPA (Overseas Publishers Association) N.V.
Published by license under
the Harwood Academic Publishers imprint,
part of The Gordon and Breach Publishing Group.
Printed in Malaysia.

### Indication for Endoscopic Resection of Submucosal Colorectal Carcinoma: Special Reference to Lymph Node Metastasis

OSAMU TSURUTA[a,*], YUICHIROH TSUJI[a], HIROSHI KAWANO[a], SHIROH MIYAZAKI[a], MASAHIDE WATANABE[a], KEITA NAKAHARA[a], HIDEO TATEISHI[a], MITSUTAKE FUJITA[a], SHIGEKI BAN[a], MICHIO SATA[a], ATSUSHI TOYONAGA[a] and MINORU MORIMATSU[b]

[a]*Department of Medicine II,* [b]*Department of Pathology, Kurume University School of Medicine, 67 Asahi-machi, Kurume City 830-0011, Japan*

*(Received 9 August 1999; Revised 27 August 1999; In final form 8 November 1999)*

**We investigated the relationship between histological factors and lymph node metastasis in 77 lesions with submucosally invasive colorectal carcinomas to establish useful criteria for lesions in which endoscopic treatment alone results in cure of malignancy. There were positive correlations between histological factors, including the level of invasion, the histologic grade, presence or absence of lymphatic invasion, presence or absence of budding, and lymph node metastasis ($p < 0.05$, $p < 0.05$, $p < 0.005$, $p < 0.01$). The presence or absence of venous invasion did not influence lymph node metastasis. Laparoscopic surgery involving lymph node dissection should be indicated for sm1 carcinoma lesions with unfavorable histological factors. In lesions diagnosed as sm2 or sm3 prior to resection, intestinal resection involving lymph node dissection by laparoscopic surgery should be directly performed without endoscopic resection.**

**In treating submucosally invasive colorectal carcinomas, the level of invasion can be clinically diagnosed, consequently endoscopic resection should be initially performed when lesions are evaluated as sm1 prior to resection. When histological investigation reveals sm1 carcinoma with histologic grade I (well-differentiated) or II (moderately-differentiated), and the absence of lymphatic invasion and budding, endoscopic treatment alone is sufficient.**

*Keywords:* Endoscopic mucosal resection, Haggitt's classification, Kudo's classification, Laparoscopic surgery, Lymph node metastasis, Submucosally invasive colorectal carcinoma

### INTRODUCTION

Advances in endoscopes and endoscopic operation techniques have increased the incidence of detection of early colorectal carcinoma. This carcinoma invades the submucosal layer or a more superficial one, and is indicated for endoscopic resection [1–15]. In particular, since endoscopic mucosal resection (EMR) was developed, superficial-type carcinoma lesions have been easily and completely

* Corresponding author. Tel.: + 81-942-31-7561. Fax: +81-942-34-2623. E-mail: tsuruta@med.kurume-u.ac.jp.

resected. Currently, endoscopic resection is most commonly performed in patients with early colorectal carcinoma [16–20].

On the other hand, laparoscopic surgery has generally been performed as minimally invasive surgery for colorectal carcinoma [21–24]. Currently, it is recommended that laparoscopic surgery should not be indicated for Dukes B or C lesions with serosal invasion, since port site recurrence may occur. However, it is indicated that laparoscopic surgery without the risk of port site recurrence should be indicated for Dukes A lesions, which invade the proper muscle layer or a more superficial site [25,26]. This procedure, differing from the standard endoscopic resection procedure, is characterized by a capacity for curative resection in lesions with lymph node metastasis, since this procedure facilitates lymph node dissection.

Therefore, endoscopic treatment is not indicated for Dukes A lesions, which invade the muscularis propria. Among carcinoma lesions that invade the submucosal layer, some cases are indicated for endoscopic treatment with radical purpose of curability, while laparoscopic surgery should be aggressively performed on lesions in which lymph node metastasis may occur. In this study, to establish useful criteria for lesions in which endoscopic treatment alone results in cure of malignancy, we examined the relationship between histological factors and lymph node metastasis in patients with early colorectal carcinoma.

### MATERIALS AND METHODS

In 77 submucosally invasive carcinoma lesions, the male-to-female ratio was 56 : 21. Mean patient ages were 62 ± 9 years for males (47–85 years) and 65 ± 11 years for females (44–78 years). Overall, the mean age was 63 ± 10 years. There was no significant difference between males and females. Tumor sites included the sigmoid colon in 29 patients, the rectum in 16, the transverse colon in 12, the ascending colon in 12, the descending colon in 7, and the cecum in 1.

The surgical resection involving lymph nodes dissection was performed at the Department of Surgery, Kurume University Hospital, between April 1995 and March 1999. As the treatment for 77 lesions of submucosally invasive carcinoma, colectomy was initially performed in 58 lesions, and colectomy following EMR in 19 lesions. EMR is a procedure in which a lesion with the peripheral normal mucosa is snared and electrically resected after elevating the lesion from the submucosal layer by infusing physiological saline solution into the submucosal layer (Fig. 1). These 77 submucosally invasive carcinomas were evaluated histopathologically at the Department of Pathology of Kurume University Hospital. Submucosally invasive carcinomas as defined for this study involved carcinomas infiltrating into the submucosal layer, but above the proper muscle layer.

Gross appearance was classified into type I (protruding type) lesions, which include Ip (pedunculated), Isp (subpedunculated), and Is (sessile) tumors, and type II (superficial type) lesions, which include IIa (superficial elevated type), IIb (superficial flat type), and IIc (superficial depressed type) tumors, according to the classification established by the Japanese Research Society for Cancer of the Colon and Rectum [27]. However, IIb type was not found in the present patients.

Five histological parameters were evaluated as follows.

(1) *Level of invasion*: When colectomy was initially performed, the submucosal layer was equally divided vertically into three sections according to the classification described by Kudo *et al.* [28], and the superficial layer was regarded as sm1, followed by the middle sm2 and the deeper sm3. In lesions for which EMR was performed, the resected submucosal layer was equally divided vertically into two sections according to the classification of Tsuruta *et al.* [29]. Carcinoma infiltration within one-half of the depth was regarded as sm1, and that beyond one-half as sm2, and positive for vertical submucosal stump as sm3 (Fig. 2). This classification seems to be possible methodology for appropriateness of determination of the invasive depth in comparing EMR specimen with colectomy specimen.

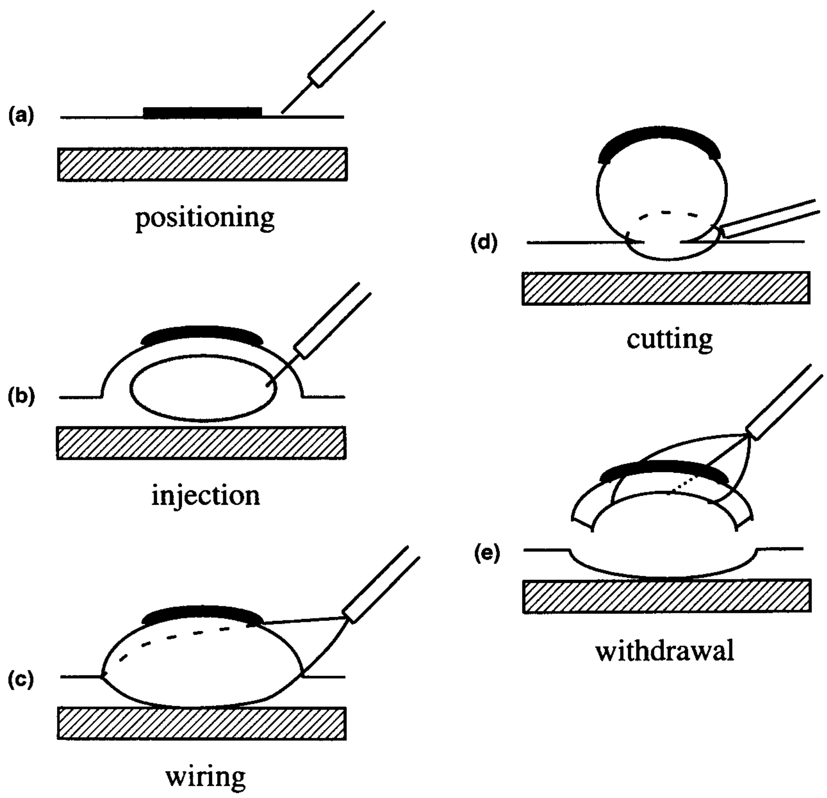

FIGURE 1 Schematic representation of endoscopic resection (EMR) procedure. (a) Optimal visualization and targeting of the lesion by endoscopy should be obtained before colonoscopic treatment. Needle forceps is used to puncture the mucosa near the lesion. (b) Physiologic saline solution is injected into the submucosa. The lesion is then elevated along with the surrounding mucosa. (c) A snare device is placed around the elevation. (d) The elevation is resected using cutting current only. (e) The resected specimen is then carefully catched by grasping forceps and withdrawn.

(2) *Histologic grade*: Histologic grade was determined by the least differentiated area of the infiltration site according to the criteria of the World Health Organization [30], and classified as grade I, II, or III. Well-differentiated adenocarcinoma was classified as grade I; moderately-differentiated adenocarcinoma as grade II; and poorly-differentiated adenocarcinoma, including signet ring cell carcinoma and mucinous adenocarcinoma as grade III.

(3) *Lymphatic invasion*: Lymphatic invasion was determined by the presence of tumor cells in the lumen covered with endothelial cells in the absence of erythrocytes.

(4) *Venous invasion*: Venous invasion was determined by the presence of tumor cells in the lumen where endothelial cells covered with the smooth muscle with elastic laminae, as detected by elastica van Gieson staining, were present.

(5) *Budding*: Budding was determined by the presence of cancer nests consisting of 5 or fewer tumor cells as described by Morodomi *et al.* [31].

The size of lesions was measured by macroscopy or dissecting microscopy of the resected specimens.

These data were statistically analyzed by Student's $t$-test, Fisher's exact test, chi-square test, or Mann–Whitney $U$-test. $p < 0.05$ was regarded as significant.

### RESULTS

With respect to gross appearance, 12 lesions were classified as Ip, 21 lesions as Isp, 2 lesions as Is,

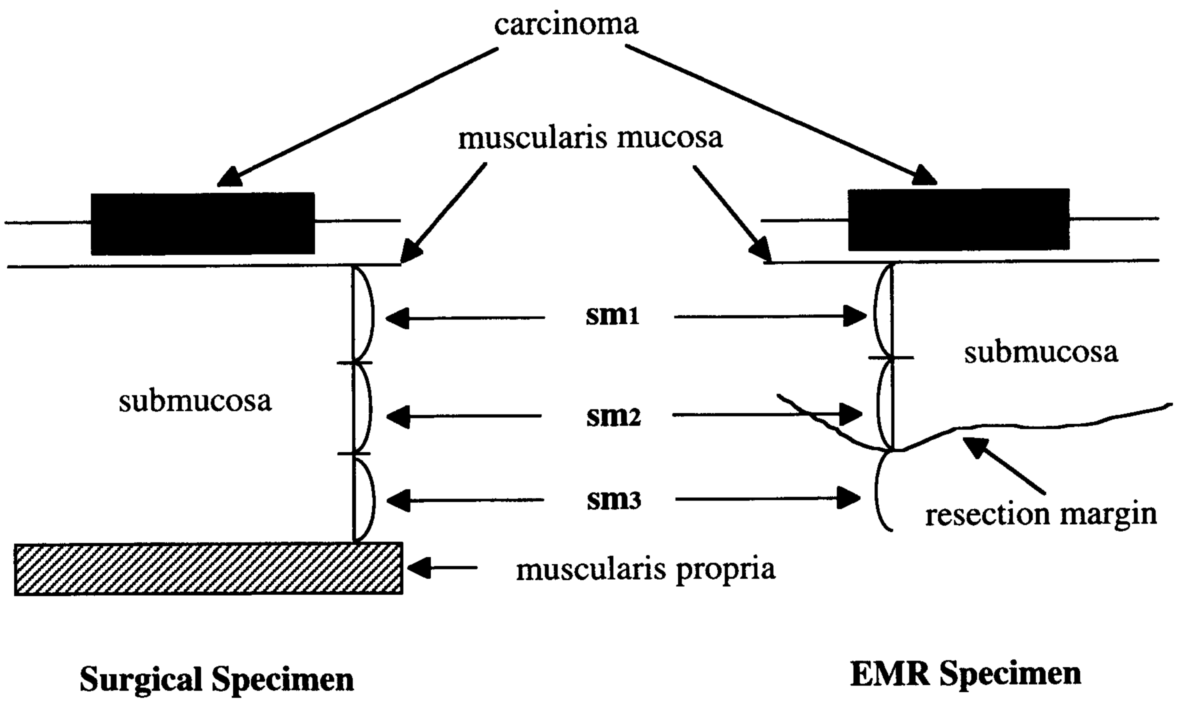

FIGURE 2 Classification of the level of invasion of submucosal invasive carcinoma. The submucosal layer was equally divided vertically into three section, the superficial layer was regarded as sm1, followed by the middle sm2 and the deeper sm3 in the case of surgical specimen. In lesions for which EMR was performed, the resected submucosal layer was equally divided vertically into two sections, carcinoma infiltration within one-half as sm2, and positive for vertical submucosal stump as sm3.

TABLE I Gross appearance and size of submucosally invasive colorectal carcinoma

| Gross appearance | Size (mm) | | | | | Total |
|---|---|---|---|---|---|---|
| | -5 | 6–10 | 11–15 | 16–20 | 21– | |
| Protruded type | | | | | | |
| Ip | 0 | 0 | 4 | 3 | 5 | 12 |
| Isp | 1 | 3 | 3 | 8 | 6 | 21 |
| Is | 0 | 0 | 1 | 0 | 1 | 2 |
| Superficial type | | | | | | |
| IIa | 0 | 6 | 4 | 6 | 5 | 21 |
| IIc | 2 | 7 | 7 | 3 | 2 | 21 |
| Total | 3 | 16 | 19 | 20 | 19 | 77 |

21 lesions as IIa, and 21 lesions as IIc. Mean tumor sizes were 22 mm (13–34 mm), 19 mm (4–38 mm), 16 mm (11–21 mm), 16 mm (7–35 mm), and 12 mm (3–30 mm), respectively. There was a significant difference in size between type I (Ip, Isp, and Is) and type IIc ($p < 0.05$) (Table I).

Treatment was examined with respect to the level of invasion. Of 19 sm1 lesions, colectomy alone was performed in 14 lesions, while colectomy following EMR was performed in 5 lesions. Of 27 sm2 lesions, colectomy alone was performed in 15 lesions, while colectomy following EMR was performed in 12 lesions. Of 31 sm3 lesions, colectomy alone was performed in 29 lesions, while colectomy following EMR was performed in 2 lesions.

### Lymph Node Metastasis Related to Histological Parameters (Table II)

Lymph node metastasis was detected in 13 (16.9%) of the 77 lesions of the submucosally invasive carcinoma examined.

*Level of Invasion* While no lymph node metastasis was detected in the 19 sm1 lesions, lymph node metastasis was detected in 4 (14.8%) of the

27 sm2 lesions, and 9 (29.0%) of the 31 sm3 lesions. There was a significant difference in metastasis with respect to level of invasion ($p < 0.05$).

*Grade III Carcinoma* Lymph node metastasis was detected in 9 (13.0%) of 69 lesions of grade I or II carcinoma. Lymph node metastasis was detected in 4 (50.0%) of 8 lesions of grade III carcinoma. There was a significant difference with respect to grade ($p < 0.05$).

*Lymphatic Invasion* While lymph node metastasis was detected in 6 (9.8%) of 61 lesions without lymphatic invasion, it was detected in 7 (43.8%) of 16 lesions with lymphatic invasion. There was a significant difference in metastasis with respect to lymphatic invasion ($p < 0.005$).

*Venous Invasion* Lymph node metastasis was detected in 10 (18.5%) of 54 lesions without venous invasion. Lymph node metastasis was detected in 3 (13.0%) of 23 lesions with venous invasion. There was no significant difference in metastasis with respect to venous invasion.

*Budding* Lymph node metastasis was detected in 2 (5.0%) of 40 lesions without budding and in 11 (29.7%) of 37 lesions with budding. There was a significant difference in metastasis with respect to budding ($p < 0.01$).

### Level of Invasion Related to Histological Parameters (Table III)

Nineteen lesions were classified as sm1, 27 lesions as sm2, and 31 lesions as sm3.

*Grade III Carcinoma* While one sm1 lesion (5.3%) was classified as grade III carcinoma, one sm2 lesion (3.7%) was classified as grade III carcinoma, and six sm3 lesions (19.4%) were classified as grade III carcinoma. There was no significant difference in grade between the three levels of invasion ($p < 0.086$).

*Lymphatic Invasion* One sm1 lesion (5.3%), three sm2 lesions (11.1%), and 12 sm3 lesions (38.7%) were positive for lymphatic invasion.

TABLE II Lymph node metastasis related to histological parameters in submucosally invasive colorectal carcinomas

| Lymph node metastasis | Level of invasion | | | Grade III carcinoma | | Lymphatic invasion | | Venous invasion | | Budding | |
|---|---|---|---|---|---|---|---|---|---|---|---|
| | sm1 | sm2 | sm3 | (−) | (+) | (−) | (+) | (−) | (+) | (−) | (+) |
| Negative | 19 | 23 | 22 | 60 | 4 | 55 | 9 | 44 | 20 | 38 | 26 |
| ($n = 64$) | (100%) | (85.2%) | (71.0%) | (87.0%) | (50.0%) | (90.2%) | (56.3%) | (81.5%) | (87.0%) | (95.0%) | (70.3%) |
| Positive | 0 | 4 | 9 | 9 | 4 | 6 | 7 | 10 | 3 | 2 | 11 |
| ($n = 13$) | (0%) | (14.8%) | (29.0%) | (13.0%) | (50.0%) | (9.8%) | (43.8%) | (18.5%) | (13.0%) | (5.0%) | (29.7%) |
| $p$-value | | < 0.05 | | < 0.05 | | < 0.005 | | NS | | < 0.01 | |

NS, not significant.

TABLE III Level of invasion related to histological parameters in submucosally invasive colorectal carcinomas

| Level of invasion | Grade III carcinoma | | Lymphatic invasion | | Venous invasion | | Budding | |
|---|---|---|---|---|---|---|---|---|
| | (−) | (+) | (−) | (+) | (−) | (+) | (−) | (+) |
| sm1 | 18 | 1 | 18 | 1 | 15 | 4 | 17 | 2 |
| ($n = 19$) | | (5.3%) | | (5.3%) | | (21.1%) | | (10.5%) |
| sm2 | 26 | 1 | 24 | 3 | 18 | 9 | 12 | 15 |
| ($n = 27$) | | (3.7%) | | (11.1%) | | (33.3%) | | (55.6%) |
| sm3 | 25 | 6 | 19 | 12 | 21 | 10 | 11 | 20 |
| ($n = 31$) | | (19.4%) | | (38.7%) | | (32.3%) | | (64.5%) |
| $p$-value | 0.086 | | < 0.005 | | NS | | < 0.005 | |

NS, not significant.

There was a significant difference in this parameter between sm1, sm2, and sm3 ($p < 0.005$).

*Venous Invasion* Four sm1 lesions (21.1%), 9 sm2 lesions (33.3%), and 10 sm3 lesions (32.3%) were positive for venous invasion. There was no significant difference.

*Budding* Two sm1 lesions (10.5%), 15 sm2 lesions (55.6%), and 20 sm3 lesions (64.5%) were positive for budding. There was a significant difference in this parameter between sm1, sm2, and sm3 ($p < 0.005$).

### CASE PRESENTATION

This is a 66-year-old male with submucosal carcinoma in the sigmoid colon.

The lesion was resected by EMR (Fig. 1). Preoperative findings by chromoendoscopy and barium enema study were shown in Fig. 3(a) and (b).

EMR specimen showed sm2, Grade II, negative lymphatic invasion, negative venous invasion and negative budding (Fig. 3(c)). Lymph node metastasis was found (Fig. 3(d)).

### DISCUSSION

Protruding lesions can be endoscopically resected by flexible endoscopic polypectomy, which was initially reported by Wolff and Shinya [32]. However, it was difficult to completely resect superficial-type lesions by this procedure. EMR [16–19] has facilitated complete resection of superficial-type lesions involving the submucosal layer and peripheral normal mucosa. For this reason, currently, endoscopic resection is aggressively indicated for all macroscopic early colorectal carcinoma lesions.

However, there is a complicating issue. When carcinoma tissues invade the submucosal layer, lymph node metastasis is detected in some lesions. Many studies have reported lymph node metastasis from submucosally invasive carcinoma. As factors, the level of invasion, histologic grade, presence or absence of lymphatic invasion, presence or absence of venous invasion, and presence or absence of budding have been indicated [1,2,4–11,13–15].

With respect to the level of invasion, Haggitt's classification [4] is commonly used in Europe and the United States. According to this classification, protruding carcinoma lesions are classified into pedunculated lesions and sessile lesions. In pedunculated lesions, the level of submucosal invasion is classified into four grades. Lesions infiltrating the polyp head are evaluated as Level 1. Lesions infiltrating the neck are evaluated as Level 2. Lesions infiltrating the stalk are evaluated as Level 3. Lesions infiltrating the intestinal wall submucosal layer below the stalk are evaluated as Level 4. Most lesions with lymph node metastasis are evaluated as Level 4. Many studies have indicated that intestinal tract resection by laparotomy involving lymph node dissection is required in Level 4 lesions [4,6,9,13]. Macroscopically, it has been reported that all sessile lesions invading the submucosal layer correspond to Level 4, and that intestinal tract resection by laparotomy involving lymph node dissection is required in all sessile submucosally invasive carcinoma lesions [4,6,9,13]. However, Nivatvongs *et al.* [9] described that the incidence of lymph node metastasis in sessile submucosally invasive carcinoma lesions was lower than that in pedunculated submucosally invasive carcinoma lesions (10%, 27%), which is different issue from previous report, although both lesions demonstrated Level 4 invasion. This difference might be explained by the nature of Haggitt's classification itself, that is, the sessile type lesion includes wide variety of carcinoma tissue invasion to the submucosal layer, but pedunculated type lesion has massive invasion as the Level 4 [4]. In this study, we used the classification of the invasion level, which may correlate with the level of invasion to the submucosal layer regardless of the gross appearance. There was a correlation between the level of invasion and lymph node metastasis ($p < 0.05$). Our classification of the invasion level may be more useful than Haggitt's classification from the perspective of availability for all gross appearance.

As for histologic grade and lymphatic invasion, there is a widely accepted concept that they have close relation to lymph node metastasis.

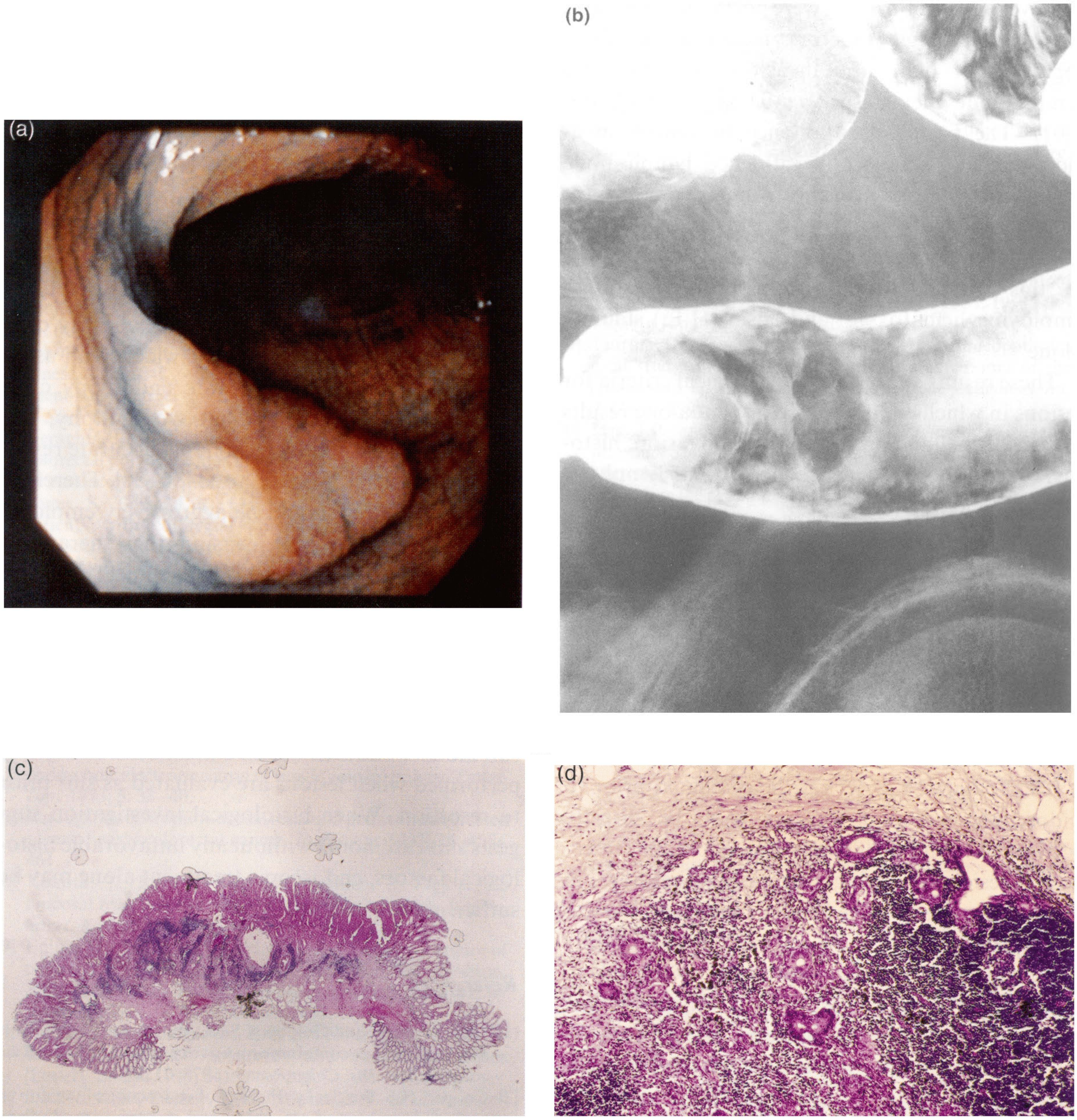

FIGURE 3 A case with submucosal carcinoma (type IIa, size 12 mm) in the sigmoid colon. (a) Chromoendoscopy reveals sessile type elevation with central depression. (b) Barium enema study shows filling defect with central barium fleckles on the flat top of the lesion. (c) EMR specimen reveals sm2, Grade II, no lymphatic invasion, no venous invasion and no budding. (d) Metastasis is found in regional lymph node.

Venous invasion is controversial as an affecting factor on lymph node metastasis, while budding in histologic findings is described as a factor to influence lymph node metastasis in the textbook edited by Japanese Research Society for Cancer of the colon and rectum.

With respect to the histologic grade, lymphatic invasion, venous invasion, and budding other than the level of invasion, there were positive correlations between histological factors, including the histologic grade, lymphatic invasion, budding, and lymph node metastasis ($p < 0.05$, $p < 0.005$, $p < 0.01$). The presence or absence of venous invasion did not statistically influence lymph node metastasis, which is different from previous report. This may have been because veins were more accurately identified by elastica van Gieson staining in this study, differing from previous studies employing hematoxylin and eosin (H.E.) staining alone.

These results suggest that histological criteria for lesions in which endoscopic treatment alone results in cure of malignancy indicate sm1 invasion, histologic grade I or II, and the absence of lymphatic invasion and budding [33].

Indication for additional colectomy after EMR must stand on strict criteria based on the histologic factors in the sm1 lesions.

When the lesions were found to be sm2 or sm3 following EMR, they must have surgery because of the great possibility of lymph node metastasis significantly suggested by the presence of multiple unfavorable histologic factors.

If histological factors can be evaluated prior to endoscopic resection, surgery may be directly performed without unnecessary endoscopic resection in lesions requiring surgery. Furthermore, in some specimens collected during endoscopic resection, histological factors cannot be sufficiently evaluated due to heat degeneration related to cauterization during resection. It is not appropriate to determine whether laparotomy should be indicated after all lesions are endoscopically resected. However, among these histological factors, only the level of invasion can be clinically diagnosed prior to resection of the lesion. It is impossible to directly and clinically diagnose other histological parameters. In this study, to examine whether other histological factors can be estimated based on the level of invasion, we investigated the relationship between the level of invasion and other four histological parameters. The level of invasion was correlated with the presence or absence of lymphatic invasion and the presence or absence of budding ($p < 0.05$, $p < 0.005$). The level of invasion slightly influenced the histologic grade ($p = 0.086$). This suggests that the histologic grade, lymphatic invasion, and budding can be predicted to some degree based on the level of invasion. Therefore, when the level of invasion is accurately diagnosed prior to resection, lesions in which endoscopic treatment alone results in cure of malignancy may be specified at a high probability.

Concerning the level of invasion of early colorectal carcinoma, along with the widespread use of barium enema examination, videoendoscopy, chromoendoscopy, endoscopic ultrasonography, and magnifying endoscopy, reasonably accurate results of diagnosis have been reported [34–38]. Therefore, these procedures should be aggressively employed to diagnose the level of invasion, then therapeutic strategies must be selected. When clinical diagnosis is sm1, EMR comes first and laparoscopic surgery with lymph node dissection comes second following careful histological examinations on EMR specimen.

In conclusion, in treating submucosally invasive carcinomas, endoscopic resection should be initially performed when lesions are evaluated as sm1 prior to resection. When histological investigation suggests sm1 carcinoma without any unfavorable histological factors, endoscopic treatment alone may be sufficient.

### *References*

[1] Nivatvongs, S. and Goldberg, S.M. Management of patients who have polyps containing invasive carcinoma removed via colonoscope. *Dis. Colon Rectum* 1978; **21**: 8–11.

[2] Cooper, H.S. Surgical pathology of endoscopically removed malignant polyps of the colon and rectum. *Am. J. Surg. Pathol.* 1983; **7**: 613–623.

[3] Langer, J.C., Cohen, Z., Taylor, B.R. *et al.* Management of patients with polyps containing malignancy removed by colonoscopic polypectomy. *Dis. Colon Rectum* 1984; **27**: 6–9.

[4] Haggitt, R.C., Glotzbach, R.E., Soffer, E.E. *et al.* Prognostic factors in colorectal carcinomas arising in adenomas: implications for lesions removed by endoscopic polypectomy. *Gastroenterology* 1985; **89**: 328–336.

[5] Cranley, J.P., Petras, R.E., Carey, W.D. *et al.* When is endoscopic polypectomy adequate therapy for colonic polyps containing invasive carcinoma? *Gastroenterology* 1986; **91**: 419–427.

[6] Cooper, H.S. The role of the pathologist in the management of patients with endoscopically removed malignant colorectal polyps. *Pathol. Annu.* 1988; **23**: 25–43.
[7] Christie, J.P. Polypectomy or colectomy? Management of 106 consecutively encountered colorectal polyps. *Am. Surg.* 1988; **54**: 93–99.
[8] Coverlizza, S., Risio, M., Ferrari, A. *et al.* Colorectal adenomas containing invasive carcinoma. Pathologic assessment of lymph node metastatic potential. *Cancer* 1989; **64**: 1937–1947.
[9] Nivatvongs, S., Rojanasakul, A., Reiman, H.M. *et al.* The risk of lymph node metastasis in colorectal polyps with invasive adenocarcinoma. *Dis. Colon Rectum* 1991; **34**: 323–328.
[10] Muller, S., Chesner, I.M., Egan, M.J. *et al.* Significance of venous and lymphatic invasion in malignant polyps of the colon and rectum. *Gut* 1989; **30**: 1385–1391.
[11] Pines, A., Bat, L., Shemesh, E. *et al.* Invasive colorectal adenomas: surgery versus colonoscopic polypectomy. *J. Surg. Oncol.* 1990; **43**: 53–55.
[12] Geraghty, J.M., Williams, C.B. and Talbot, I.C. Malignant colorectal polyps: venous invasion and successful treatment by endoscopic polypectomy. *Gut* 1991; **32**: 774–778.
[13] Kyzer, S., Bégin, L.R., Gordon, P.H. *et al.* The care of patients with colorectal polyps that contain invasive adenocarcinoma. Endoscopic polypectomy or colectomy? *Cancer* 1992; **70**: 2044–2050.
[14] Cooper, H.S., Deppisch, L.M., Gourley, W.K. *et al.* Endoscopically removed malignant colorectal polyps: clinicopathologic correlations. *Gastroenterology* 1995; **108**: 1657–1665.
[15] Hackelsberger, A., Frühmorgen, P., Weiler, H. *et al.* Endoscopic polypectomy and management of colorectal adenomas with invasive carcinoma. *Endoscopy* 1995; **27**: 153–158.
[16] Deyhle, P., Largiader, F., Jenny, S. *et al.* A method for endoscopic electroresection of sessile colonic polyps. *Endoscopy* 1973; **5**: 38–40.
[17] Kudo, S. Endoscopic mucosal resection of flat and depressed types of early colorectal cancer. *Endoscopy* 1993; **25**: 455–461.
[18] Yokota, T., Sugihara, K. and Yoshida, S. Endoscopic mucosal resection for colorectal neoplastic lesions. *Gastrointest. Endosc.* 1995; **42**: 600–601.
[19] Fujita, M., Tsuruta, O., Ikeda, H. *et al.* Local recurrence of colorectal tumors after endoscopic mucosal resection: evaluation of the lateral margin of resected specimen by stereomicroscopy. *Int. J. Oncol.* 1997; **11**: 533–538.
[20] Alexander, R.J.T., Jaques, B.C. and Mitchell, K.G. Laparoscopically assisted colectomy and wound recurrence [Letter]. *Lancet* 1993; **341**: 249–250.
[21] Zucker, K.A., Pitcher, D.E., Martin, D.T. *et al.* Laparoscopic-assisted colon resection. *Gr. J. Surg.* 1994; **8**: 12–18.
[22] Wexner, S.D., Reissman, P., Pfeifer, J. *et al.* Laparoscopic colorectal surgery: analysis of 140 cases. *Surg. Endosc.* 1996; **10**: 133–136.
[23] Bokey, E.L., Moore, W.E., Keating, J.P. *et al.* Laparoscopic resection of the colon and rectum for cancer. *Br. J. Surg.* 1997; **84**: 822–825.
[24] Boulez, J., Espalieu, Ph., Fontaumard, E. *et al.* Laparoscopic colo-rectal surgery: analysis of 113 cases. *Hepatogastroenterology* 1997; **44**: 40–44.
[25] Kitamura, K., Taniguchi, H., Yamaguchi, T. *et al.* Clinical outcome of surgical treatment for invasive early colorectal cancer in Japan. *Hepatogastroenterology* 1997; **44**: 108–115.
[26] Adachi, Y., Sato, K., Shiraishi, N. *et al.* Tumor size of colorectal cancer: indication for laparoscopic surgery. *Surg. Laparosc. Endosc.* 1998; **8**: 269–272.
[27] Japanese Research Society for Cancer of the Colon and Rectum. General rules for clinical and pathological studies on cancer of the colon, rectum and anus. Part I. Clinical classification. *Jpn. J. Surg.* 1983; **13**: 557–573.
[28] Kudo, S., Soga, J., Tamamoto, M. *et al.* Treatment of colorectal sm-carcinomas. *Stomach Intest.* 1984; **19**: 1349–1355 (in Japanese with English abstract).
[29] Tsuruta, O., Toyonaga, A., Ikeda, H. *et al.* Clinicopathological study of superficial-type invasive carcinoma of the colorectum: special reference to lymph node metastasis. *Int. J. Oncol.* 1998; **10**: 769–775.
[30] Jass, J.R. and Sobin, L.H. *WHO Histological Typing of Intestinal Tumors.* Berlin, Germany: Springer-Verlag, 1989.
[31] Morodomi, T., Isomoto, H., Shirouzu, K. *et al.* An index for estimating the probability of lymph node metastasis in rectal cancers. Lymph node metastasis and the histopathology of actively invasive regions of cancer. *Cancer* 1989; **63**: 539–543.
[32] Woff, W.I. and Shinya, H. Polypectomy via the fiberoptic colonoscope. Removal of neoplasms beyond reach of the sigmoidoscope. *N. Engl. J. Med.* 1973; **288**: 329–332.
[33] Kodaira, S., Yao, T., Nakamura, K. *et al.* Submucosal invasive carcinoma of the colon and rectum with metastasis-analysis of 1917 cases focused on sm invasion. *Stomach Intest.* 1994; **29**: 1137–1149 (in Japanese with English abstract).
[34] Watari, J., Saitoh, Y., Orii, Y. *et al.* Early nonpolypoid colorectal cancer: radiographic diagnosis of depth of invasion. *Radiology* 1997; **205**: 67–74.
[35] Saitoh, Y., Obara, T., Watari, J. *et al.* Invasion depth diagnosis of depressed type early colorectal cancers by combined use of videoendoscopy and chromoendoscopy. *Gastrointest. Endosc.* 1998; **48**: 362–370.
[36] Saitoh, Y., Obara, T., Einami, K. *et al.* Efficacy of high-frequency ultrasound proves for the preoperative staging of invasion depth in flat and depressed colorectal tumors. *Gastrointest. Endosc.* 1996; **44**: 34–39.
[37] Tsuruta, O., Kawano, H., Fujita, M. *et al.* Usefulness of the high-frequency ultrasound prove in pretherapeutic staging of superficial-type colorectal tumors. *Int. J. Oncol.* 1998; **13**: 677–684.
[38] Kudo, S., Tamura, F., Nakajima, T. *et al.* Diagnosis of colorectal tumorous lesions by magnifying endoscopy. *Gastrointest. Endosc.* 1996; **44**: 8–11.

